# Effects of dietary n-6/n-3 PUFA ratio on growth performance and lipid metabolism in nursery pigs

**DOI:** 10.3389/fvets.2025.1643724

**Published:** 2025-09-09

**Authors:** Junjie Guo, Xiaoqian Chen, Huiling Zhu, Kan Xiao, Yanbing Zhang, Shiwei Zhao, Guoshun Chen, Yulan Liu

**Affiliations:** 1 College of Animal Science and Technology, Gansu Agricultural University, Lanzhou, China; 2 Hubei Key Laboratory of Animal Nutrition and Feed Science, Wuhan Polytechnic University, Wuhan, China; 3 Shandong Zhongmu Feed Technology Co., Ltd., Binzhou, Shandong, China; 4 Shandong Crelipids Biotechnology Co., Ltd., Binzhou, Shandong, China

**Keywords:** n-6 PUFA, n-3 PUFA, fatty acid, lipid metabolism, nursery pigs

## Abstract

The proportion of n-6 and n-3 polyunsaturated fatty acid (PUFA) in commercial pig feed is severely unbalanced. This study was conducted to investigate the effects of different n-6/n-3 PUFA ratios on growth performance and lipid metabolism of nursery pigs. A total of 240 nursery pigs (Duroc × Large White × Landrace) were fed diets with different n-6/n-3 PUFA ratios, including 10:1, 5:1, 3:1, and 1.5:1. Pigs fed diet with n-6/n-3 PUFA ratio of 1.5:1 or 3:1 had optimum average daily gain and feed to gain ratio (*p* < 0.05). The levels of serum lipids including total cholesterol, triglyceride, high density lipoprotein and low density lipoprotein were the lowest in pigs fed diet with n-6/n-3 PUFA ratio of 1.5:1 (*p* < 0.05). The concentrations of serum insulin, adiponectin and leptin were the highest in pigs fed diet with n-6/n-3 PUFA ratio of 3:1 (*p* < 0.05). Pigs fed diet with n-6/n-3 PUFA ratio of 3:1 had the highest abundance of genes associated with fatty acid absorption and transportation (*FATP4*, and *PPARγ*), synthesis and storage (*FAS* and *GPAT*) and degradation (*ATGL*, *HSL*, and *MAGL*) in intestine (*p* < 0.05). Pigs fed diet with n-6/n-3 PUFA ratio of 1.5:1 had the lowest abundance of genes associated with fatty acid absorption (*CD36* and *FABP4*), synthesis and storage (*ACC*, *FAS*, *ACLY*, *PAP*, *AGPAT*, and *GPAT*) and degradation (*CPT1* and *HSL*) in gastrocnemius muscle (*p* < 0.05). The mRNA expression of genes associated with fatty acid metabolism (*FATP2*, *FATP5*, *FABP1*, *FABP4*, *LPL*, *ACS*, *ACLY*, *AGPAT*, *GPAT*, *CPT1*, *ATGL*, and *MAGL*) was up-regulated in liver and subcutaneous fat of pigs fed diet with n-6/n-3 PUFA ratios of 1.5:1–5:1 (*p* < 0.05). In summary, diets with lower n-6/n-3 PUFA ratios improve growth performance, reduce blood lipids, facilitate lipid metabolism in intestine, liver and subcutaneous fat, and inhibit fatty acid absorption, synthesis and storage in gastrocnemius muscle in pigs.

## Introduction

Fatty acids are one of the body’s main sources of energy. Essential fatty acids, including n-3 polyunsaturated fatty acids (PUFA) and n-6 PUFA, cannot be synthesized from scratch by animal bodies and must be obtained from the diet to meet the demand. These two types of PUFA cannot convert to each other and have opposite physiological functions (1). It is well established that the oxidized lipids produced by n-6 PUFA can promote the release of inflammatory factors more than those produced by n-3 PUFA (2, 3). In addition, intake of a diet containing a high proportion of n-6/n-3 PUFA is associated with the development of diseases such as obesity, diabetes and angiocardiopathy (4, 5). It has been reported that dietary n-6 and n-3 PUFA have become severely unbalanced in humans over the past few decades, reaching 20:1 instead of 1:1 that was during evolution (6). Current commercial swine diets are rich in n-6 PUFA, with an n-6/n-3 PUFA ratio of 10:1, which is also considered not to be the optimal n-6/n-3 PUFA ratio (7). Among plant-derived oils, linseed oil exhibits the highest concentration of n-3 PUFAs, making it the most efficient option for modulating the dietary n-6/n-3 PUFA ratio (8). Therefore, an appropriate n-6/n-3 PUFA ratio is particularly important for growth and development of animals.

Animal growth and development are closely related to fat metabolism. Both n-6 PUFA and n-3 PUFA belong to long-chain PUFA, which are absorbed and metabolized slowly in mammals. The metabolic process of these fatty acids includes absorption and transportation, synthesis and storage, and decomposition and release of fatty acids (9). Free fatty acids are absorbed and transported into cells by the fatty acid transport protein (FATP) family and fatty acid translocase (FAT; CD36), and then transported to the smooth endoplasmic reticulum by fatty acid-binding protein (FABP) for fatty acid synthesis (10). The synthesis of fatty acids mainly includes monoacylglycerol acyltransferase (MGAT) pathway and glycerol 3 phosphate (G3P) pathway (11). The triglycerides (TAG) in gut are mainly synthesized by sn-2 monoacylglycerol and fatty acyl-CoA catalyzed by MGAT, accounting for about 80% (10). The G3P pathway mainly occurs in liver, skeletal muscle and adipose tissue cells, involving in key enzymes such as glycerol 3 phosphate acyltransferase (GPAT), acylglycerol 3 phosphate acyltransferase (AGPAT), phosphatidic acid phosphatase (PAP) and diacylglycerol acyltransferases (DGAT) (10, 12). Triglycerides stored in lipid droplets release three fatty acids under hydrolysis of adipose triglyceride lipase (ATGL), hormone sensitive lipase (HSL) and monoacylglycerol lipase (MAGL) (9). The *β*-oxidation process in mitochondria is the main mode of complete fat degradation, and the main rate-limiting enzyme in this process is carnitine palmitoyl transferase 1 (CPT1) (13). Previous studies have shown that diet enriched with n-3 PUFA can affect fatty acid metabolism in liver of Polish Landrace pig (14). It is well known that excess n-6 PUFA competes with n-3 PUFA in the same group of desaturases, elongases, and oxygenases to complete metabolism and is associated with the presence or development of obesity, inflammation, lipid toxicity, imbalance of lipid metabolism, and abnormal lipid accumulation (15, 16). However, limited data on how n-6/n-3 PUFA ratio affects the metabolism of fatty acids in the gut, liver, skeletal muscle, and adipose tissue.

To the best of our knowledge, while previous studies have extensively investigated fatty acid metabolism in finishing pigs, gestating sows, lactating sows, and suckling piglets, the nursery pig stage remains unexplored (1, 7, 17–20). The nursery phase represents a critical developmental window for pigs. The survival rate and growth performance of piglets in this phase significantly influence the economic efficiency of swine production. Thus, the purpose of this study was to determine the effect of different proportions of dietary n-6/n-3 PUFA on growth performance and lipid metabolism in nursery pigs. Our study will provide a new reference for the rational utilization of n-6 and n-3 PUFA in diets and for understanding the metabolism of fatty acids in nursery pigs.

## Materials and methods

### Animals and experimental design

All experimental procedures for nursery pigs in the present study were approved by the Animal Care and Use Committee of Wuhan Polytechnic University (Wuhan, China) (WPU202306006). A total of 240 male cross-bred pigs (Duroc × Large White × Landrace) with a similar initial weight of 7.89 ± 0.18 kg were obtained from Aodeng Agriculture and Animal Husbandry Technology Co., Ltd., Hubei, China. They were assigned to 4 groups using a randomized complete block design based on body weight, each consisting of 6 replicates of 10 pigs each. Ten piglets were raised in a 3 × 3 m pen with plastic slatted flooring to allow for natural waste removal. The pigs were fed one of corn-soybean meal-based diets containing 3% oil as follows: 3% soybean oil (Yihai Kerry Arawana Holdings Co., Ltd., Wuhan, China), 2.25% soybean oil and 0.75% linseed oil (Yihai Kerry Arawana Holdings Co., Ltd., Wuhan, China), 1.5% soybean oil and 1.5% linseed oil, and 3% linseed oil, resulting in dietary n-6/n-3 ratios of approximately 10:1, 5:1, 3:1, and 1.5:1, respectively. The composition and nutritional levels of the diets are shown in Table 1. The fatty acid composition of the diets is listed in Table 2. All pigs had *ad libitum* access to experimental diets and water for 28 days. On days 14 and 28 of the experiment, body weight of each piglet and feed intake of each pen were recorded, and average daily gain (ADG), average daily feed intake (ADFI) and feed to gain ratio (F/G) in each pen were calculated (*n* = 6).

**Table 1:** Composition and nutrient contents of the diets (as fed basis).

Item	n-6:n-3 PUFA ratio			
	10:1	5:1	3:1	1.5:1
Ingredients (%)				
Corn	38.60	38.60	38.60	38.60
Extruded corn	10.00	10.00	10.00	10.00
Broken rice	14.00	14.00	14.00	14.00
Soybean meal	19.50	19.50	19.50	19.50
Extruded soybean	6.33	6.33	6.33	6.33
Fermented soybean meal	2.09	2.09	2.09	2.09
Fish meal	1.78	1.78	1.78	1.78
Whey power	1.16	1.16	1.16	1.16
Soybean oil	3.00	2.25	1.50	0.00
Flaxseed oil	0.00	0.75	1.50	3.00
Limestone	0.57	0.57	0.57	0.57
Calcium hydrophosphate	1.54	1.54	1.54	1.54
Salt	0.44	0.44	0.44	0.44
DL-methionine (98% Methionine)	0.06	0.06	0.06	0.06
L-Lysine (98% Lysine)	0.34	0.34	0.34	0.34
Vitamin and mineral premix^a^	0.68	0.68	0.68	0.68
Nutrient composition^b^				
Digestible energy, kcal/kg	3,544	3,544	3,544	3,544
Crude protein	19.40	19.40	19.40	19.40
Calcium	0.75	0.75	0.75	0.75
Total phosphorus	0.70	0.70	0.70	0.70
Non-phytate phosphorus	0.43	0.43	0.43	0.43
Lysine	1.35	1.35	1.35	1.35
Methionine	0.38	0.38	0.38	0.38
Threonine	0.70	0.70	0.70	0.70

^a^Vitamin and mineral premix provided per kg of diet: Vitamin A, 2,750 μg; Vitamin D_3_, 63 μg; DL-*α*-tocopheryl acetate, 20 mg; Vitamin K, 3 mg; Vitamin B_12_, 18 mg; Vitamin B_1_, 1.5 mg; Vitamin B_2_, 4 mg; Vitamin B_6_, 3 mg; pantothenic acid, 15 mg; Niacin, 40 mg; Choline chloride, 400 mg; Folic acid, 700 mg; Biotin, 100 mg; Zn, 80 mg (ZnSO_4_·7H_2_O); Mn, 20 mg (MnSO_4_·5H_2_O); Fe, 82 mg (FeSO_4_·H_2_O); Cu, 25 mg (CuSO_4_·5H_2_O); I, 0.47 mg (KI); Se, 0.30 mg (Na_2_SeO_3_).

^b^The nutrient contents were all calculated values.

**Table 2:** Fatty acid composition of diets (% of total fatty acids).

Fatty acids	n-6:n-3 PUFA ratio			
	10:1	5:1	3:1	1.5:1
C14:0	0.21	0.21	0.20	0.20
C16:0	13.82	13.33	12.76	11.90
C16:1	0.28	0.29	0.29	0.27
C17:0	0.20	0.16	0.18	0.15
C18:0	3.59	3.62	3.73	3.81
C18:1 (n-9)	24.02	23.30	22.65	21.20
C18:2 (n-6)	50.86	47.29	43.60	37.61
C18:3 (n-3)	4.62	8.74	13.63	22.20
C20:0	0.42	0.38	0.36	0.31
C20:1	0.28	0.63	0.59	0.60
C21:0	0.12	0.11	0.10	0.09
C20:3 (n-3)	0.03	0.12	0.12	0.04
C22:0	0.45	0.40	0.39	0.31
C20:5 (n-3)	0.09	0.09	0.11	0.11
C23:0	0.11	0.14	0.08	0.07
C24:0	0.29	0.25	0.23	0.22
C22:6 (n-3)	0.35	0.35	0.34	0.34
Total (n-6) PUFA	50.86	47.29	43.60	37.61
Total (n-3) PUFA	5.08	9.30	14.21	22.69
(n-6)/(n-3)^a^	10.01	5.08	3.07	1.66

^a^(n-6)/(n-3) ratio calculated as (C18:2, n-6)/(C18:3 + C20:3 + C20:5 + C22:6, n-3). The fatty acids which content was lower than 0.1% of total fatty acids are not presented.

### Sample collection

On day 28, one pig with average body weight was selected from each replicate pen to collect blood from the anterior vena cava with a 10 mL vacuum tube, and then slaughtered under anesthesia. Subsequently, approximately 10 g tissue samples were collected from the jejunal mucosa (middle section), the left and right outer lobes of the liver, gastrocnemius muscle, and subcutaneous fat located in the gastrocnemius muscle. All samples were immediately snap-frozen in liquid nitrogen and stored at −80 °C for subsequent analysis.

### Fatty acid composition analysis

The fatty acid composition of the feed was determined using a modified one-step derivatization method based on Lepage and Roy (21). Briefly, samples were extracted with a mixture of acetyl chloride-methanol (1:10 v/v). Then, total fat was converted into fatty acid methyl esters (FAMEs) and determined by Agilent 7890b gas chromatography, which was equipped with a flame ionization detector (Agilent Technologies, Inc., Santa Clara, California, USA). A HP-88 fused silica capillary column (Agilent Technologies, Inc., Santa Clara, California, USA) was used to separate FAMEs. Fatty acids were identified by matching both retention times and peak areas with corresponding authentic standards. The relative content of each fatty acid was calculated as a percentage of the total fatty acid content.

### Serum lipid analysis

All blood samples were centrifuged at 3000 g for 10 min to collect serum. The contents of total cholesterol (TC), triglyceride (TG), high density lipoprotein (HDL), low density lipoprotein (LDL), and glucose in serum were determined by HITEC 7100 automatic biochemical analyzer.

### Serum hormone measurement

The concentrations of insulin, adiponectin and leptin in the serum were measured by commercial enzyme-linked immunosorbent assay (ELISA) kits (Quanzhou Ruixin Biotechnology Co., Ltd., Fujian, China). All experimental processes were performed according to the manufacturer’s instructions.

### mRNA abundance determination

Total RNA was extracted from the jejunal mucosa, liver, gastrocnemius muscle and subcutaneous fat using the trizol reagent (Nanjing Vazyme Biotech Co., Ltd., Nanjing, China). The concentration and purity of RNA were determined by NanoDrop2000 spectrophotometer (Thermo Fisher Scientific, Inc.). The degradation of RNA was detected by 1% agarose gel electrophoresis. The synthesis of cDNA and quantitative PCR (*n* = 3) were carried out by PrimeScript® RT kit (Nanjing Vazyme Biotech Co., Ltd., Nanjing, China) and SYBR® Premix Ex Taq™ (Tli RNaseH Plus) qPCR kit (Nanjing Vazyme Biotech Co., Ltd., Nanjing, China), respectively. The primer sequences of target genes 5′ and 3′ are listed in Table 3. *β*-actin, a house-keeping gene, was used as an internal control to normalize the expression of target genes. As Livak et al. previously reported, the relative expression of target genes was calculated according to the 2^-△△CT^ method (22).

**Table 3:** The primer sequences of the target genes.

Gene	Primer sequence	Amplification length	Serial number
*β-actin*	F: TGCGGGACATCAAGGAGAAG	216	NM_001167795.1
	R: AGTTGAAGGTGGTCTCGTGG		
*CD36*	F: AGCACTTACTTGGATGTTGA	143	NM_001044622.1
	R: CAGAGGATAGGCACGATATAG		
*FATP2*	F: AGAATACAGGACACCATTGA	143	NM_001278777.1
	R: TCAGTCATAGGCACATACG		
*FATP4*	F: CCTGGTGTACTACGGATTC	230	XM_013993903.2
	R: GCTGGTTGAGGAGGTATC		
*FATP5*	F: CAGGTAAGTCGCCAGATG	100	XM_021097370.1
	R: CGGAGAGGTACTTGTAAGG		
*FABP1*	F: GACGAACTCATCCAGAAGG	142	NM_001004046.2
	R: TCTCCATCTCACACTCCTC		
*FABP4*	F: GTCAAGAGCACCATAACCT	114	NM_001002817.1
	R: ACATTCCACCACCAACTTAT		
*PPARγ*	F: ACAGCGACCTGGCGATATTT	117	NM_214379.1
	R: GCAGCTCCAAGGCTTGCA		
*PPARα*	F: GCCCAAGTTTGACTTCGCCATGAA	151	NM_001044526.1
	R: ATGCACGATACCCTCCTGCATTCT		
*SREBP1*	F: GCTCCTCCATCAATGACAAG	116	NM_214157.1
	R: CTGAAGGAAGCGGATGTAGT		
*LPL*	F: GCAGGAAGTCTGACCAATAA	294	NM_214286.1
	R: CTTCACCAGCTGGTCCACAT		
*ACC*	F: CTCAAGTCACCAAGAAGAATC	231	XM_021066238.1
	R: CAATAGCCGATAGGAAGATAGA		
*ACS*	F: GTAATTGGTGGACAGAACATC	187	XM_013986330.2
	R: ACTCTCCTGCTTGTAACTTC		
*FAS*	F: GGTTAAGAGTGAATACGATGAC	205	NM_213839.1
	R: AGGCAGTGATTGTGACATT		
*ACLY*	F: CGAGGTCTTCAAGGAGGA	107	NM_001257276.1
	R: CCATCAGGCACATCTCAAT		
*PAP*	F: CGACATTGACGGAACCAT	137	NM_001257276.1
	R: AGCAGTAGAGGAACTTATACC		
*AGPAT*	F: ATCCTCTTCCTGGCTGTG	175	NM_001033008.2
	R: TGGAGACGACGACATAGG		
*GPAT*	F: CGATAATACAGTTGGTCAGAG	200	XM_005671462.3
	R: CTCAGTGGTAAGTCCTATCAT		
*DGAT*	F: GTTCAGTTCAGACAGTGGTT	110	NM_214051.1
	R: CGTACTTGATGAGGTTCTCTA		
*CPT1*	F: TCATCAAGGAGGTAGGTAGG	109	NM_001129805.1
	R: TCAGTGGTCAACAGTGTATG		
*ATGL*	F: ACCTTCATTCCCGTGTACTG	110	XM_021076533.1
	R: ATGGTGCTCTTGAGTTCGTA		
*HSL*	F: CTGGAATATCACCGAGATTG	208	NM_214315.3
	R: ACGCAGGTCATAGGAGAT		
*MAGL*	F: GGTGGACCTCTACAATGC	286	XM_021068336.1
	R: CTCACGGAAGACGGAATC		

### Statistical analysis

All the experimental data were analyzed using one-way ANOVA in SPSS Statistics 20 software (SPSS Inc., USA), followed by Duncan’s multiple comparisons. Furthermore, the linear and quadratic effects of decreasing dietary n-6/n-3 PUFA ratio were analyzed using orthogonal comparisons. All data in the tables are expressed as means ± SE. Differences were considered to be statistically significant at *p* < 0.05 and trends at 0.05 ≤ *p* < 0.10.

## Results

### Effect of different n-6/n-3 PUFA ratios on growth performance in nursery pigs

With the decrease of dietary n-6/n-3 PUFA ratios, ADG during 1–14 days (linear, *p* < 0.05; quadratic, *p* < 0.10), and ADG during 1–28 days (linear, *p* < 0.10) were increased, and F/G ratio during 1–14 (linear, *p* < 0.01; quadratic, *p* < 0.01), 14–28 (linear, *p* < 0.01; quadratic, *p* < 0.01) and 1–28 days (linear, *p* < 0.001; quadratic, *p* < 0.001) was decreased (Table 4). However, ADFI was not affected by n-6/n-3 PUFA ratios. Pigs fed diet with n-6/n-3 PUFA ratio of 3 had higher ADG during 1–14 days (*p* < 0.05) and lower F/G ratio during 1–28 days (*p* < 0.001) than those of 10:1 and 5:1. Pigs fed diet with n-6/n-3 PUFA ratio of 1.5:1 had the lowest F/G ratio during 1–14 (*p* < 0.01), 14–28 (*p* < 0.05) and 1–28 days (*p* < 0.001).

**Table 4:** Effect of different n-6/n-3 PUFA ratio on growth performance in nursery pigs.

Item	n-6:n-3 PUFA ratio				*p*-value	
	10:1	5:1	3:1	1.5:1	Linear	Quadratic
Day 1 body weight, kg	7.90 ± 0.40	7.85 ± 0.40	7.90 ± 0.42	7.90 ± 0.40	0.976	0.997
Day 14 body weight, kg	11.3 ± 0.42	11.2 ± 0.53	11.8 ± 0.58	11.6 ± 0.44	0.447	0.754
Day 28 body weight, kg	18.1 ± 0.91	17.8 ± 0.86	19.0 ± 0.88	19.2 ± 0.76	0.249	0.501
Days 1–14						
Average daily gain, g/d	242 ± 7^b^	241 ± 10^b^	278 ± 13^a^	268 ± 7^ab^	0.020	0.067
Average daily feed intake, g/d	494 ± 19	481 ± 13	527 ± 21	471 ± 20	0.805	0.551
Feed to gain ratio, g/g	2.05 ± 0.08^a^	2.00 ± 0.03^a^	1.90 ± 0.04^ab^	1.76 ± 0.05^b^	0.001	0.002
Days 14–28						
Average daily gain, g/d	488 ± 38	471 ± 24	514 ± 26	538 ± 27	0.142	0.272
Average daily feed intake, g/d	872 ± 52	857 ± 51	899 ± 38	892 ± 51	0.617	0.882
Feed to gain ratio, g/g	1.80 ± 0.05^a^	1.82 ± 0.03^a^	1.75 ± 0.03^ab^	1.66 ± 0.01^b^	0.003	0.004
Days 1–28						
Average daily gain, g/d	365 ± 19	356 ± 17	396 ± 17	403 ± 16	0.058	0.157
Average daily feed intake, g/d	683 ± 32	669 ± 31	713 ± 29	682 ± 36	0.772	0.926
Feed to gain ratio, g/g	1.87 ± 0.02^a^	1.88 ± 0.02^a^	1.80 ± 0.02^b^	1.69 ± 0.02^c^	<0.001	<0.001

All values are presented as mean ± standard error. *n* = 6; ^a,b,c^Different superscripts within a row are significantly different (*p* < 0.05).

### Effect of different n-6/n-3 PUFA ratios on contents of serum lipids in nursery pigs

With the decrease of dietary n-6/n-3 PUFA ratio, the contents of TC (*p* < 0.01; *p* < 0.05), TG (*p* < 0.01; *p* < 0.01), HDL (*p* < 0.05; *p* < 0.05) and LDL (*p* < 0.05; *p* < 0.10) in serum were decreased linearly and quadratically, respectively (Table 5). Furthermore, compared with diets with n-6/n-3 PUFA ratios of 10:1, 5:1 and 3:1, pigs fed diet with n-6/n-3 PUFA ratio of 1.5:1 had the lowest contents of TC, TG, HDL and LDL in serum (*p* < 0.05).

**Table 5:** Effect of different n-6/n-3 PUFA ratio on serum biochemical indices in nursery pigs.

Item	n-6:n-3 PUFA ratio				*p*-value	
	10:1	5:1	3:1	1.5:1	Linear	Quadratic
Total cholesterol, mmol/L	2.32 ± 0.08^a^	2.13 ± 0.09^ab^	2.21 ± 0.04^a^	1.97 ± 0.05^b^	0.006	0.023
Triglyceride, mmol/L	0.65 ± 0.08^ab^	0.70 ± 0.05^a^	0.53 ± 0.05^bc^	0.44 ± 0.03^c^	0.004	0.009
High density lipoprotein, mmol/L	0.95 ± 0.04^a^	0.92 ± 0.04^ab^	0.94 ± 0.04^a^	0.81 ± 0.03^b^	0.037	0.050
Low density lipoprotein, mmol/L	1.01 ± 0.06^a^	0.86 ± 0.05^ab^	0.89 ± 0.05^ab^	0.80 ± 0.04^b^	0.016	0.051
Glucose, mmol/L	2.98 ± 0.58	2.78 ± 0.24	3.47 ± 0.40	3.42 ± 0.21	0.251	0.516

All values are presented as mean ± standard error. *n* = 6; ^a,b,c^Different superscripts within a row are significantly different (*p* < 0.05).

### Effect of different n-6/n-3 PUFA ratios on serum hormone levels in nursery pigs

With the decrease of dietary n-6/n-3 PUFA ratios, the concentrations of insulin (quadratic, *p* < 0.01), adiponectin (quadratic, *p* < 0.05) and leptin (linear, *p* < 0.10; quadratic, *p* < 0.05) in the serum was increased (Table 6). Pigs fed diet with n-6/n-3 PUFA ratio of 3:1 had the highest concentrations of insulin, adiponectin and leptin in the serum compared with diets with n-6/n-3 PUFA ratios of 10:1, 5:1 and 1.5:1 (*p* < 0.05).

**Table 6:** Effect of different n-6/n-3 PUFA ratio on serum hormone levels in nursery pigs.

Item	n-6:n-3 PUFA ratio				*p*-value	
	10:1	5:1	3:1	1.5:1	Linear	Quadratic
Insulin, mIU/L	1.99 ± 0.35^b^	4.63 ± 0.53^b^	9.60 ± 2.06^a^	1.99 ± 0.20^b^	0.511	0.002
Adiponectin, mg/L	8.27 ± 0.67^b^	9.17 ± 1.52^b^	12.95 ± 1.19^a^	7.58 ± 0.63^b^	0.779	0.050
Leptin, μg/L	0.01 ± 0.01^b^	0.46 ± 0.11^ab^	1.18 ± 0.51^a^	0.66 ± 0.22^ab^	0.057	0.044

All values are presented as mean ± standard error. *n* = 6; ^a,b^Different superscripts within a row are significantly different (*p* < 0.05).

### Effect of different n-6/n-3 PUFA ratios on gene expression related to fatty acid metabolism in jejunum, liver, gastrocnemius muscle and subcutaneous fat

With the decrease of dietary n-6/n-3 PUFA ratio, the mRNA expression of *FATP2* (*p* < 0.05; *p* < 0.10) and *FATP5* (*p* < 0.01; *p* < 0.01) in jejunum was decreased linearly and quadratically, respectively (Table 7). With the decrease of dietary n-6/n-3 PUFA ratios, the mRNA expression of *FATP4* (*p* < 0.01), peroxisome proliferator activated receptor (*PPAR*)*γ* (*p* < 0.10), *FAS* (*p* < 0.10), *GPAT* (*p* < 0.10), *ATGL* (*p* < 0.05), and *HSL* (*p* < 0.01) in jejunum was increased quadratically. Pigs fed diet with n-6/n-3 PUFA ratio of 3:1 had the highest mRNA expression of *FATP4*, *ATGL*, and *HSL* in jejunum compared with diets with n-6/n-3 PUFA ratios of 10:1, 5:1 and 1.5:1 (*p* < 0.05). Compared with diet with n-6/n-3 PUFA ratio of 10:1, pigs fed diets with n-6/n-3 PUFA ratios of 5:1 and 1.5:1 had the lowest mRNA expression of *FATP2* and *FATP5* in jejunum (*p* < 0.05).

**Table 7:** Effect of different n-6/n-3 PUFA ratio on gene expression related to fatty acid metabolism in jejunum.

Item	n-6:n-3 PUFA ratio				*p*-value	
	10:1	5:1	3:1	1.5:1	Linear	Quadratic
Absorption and transportation						
*FATP2*	1.00 ± 0.17^a^	0.62 ± 0.03^b^	0.73 ± 0.16^ab^	0.50 ± 0.06^b^	0.017	0.052
*CD36*	1.00 ± 0.18	3.59 ± 1.90	2.83 ± 1.02	1.57 ± 0.21	0.852	0.217
*FABP1*	1.00 ± 0.13	1.64 ± 0.39	1.86 ± 0.85	0.78 ± 0.14	0.846	0.202
*FATP4*	1.00 ± 0.21^b^	3.07 ± 0.56^a^	3.44 ± 0.72^a^	2.10 ± 0.61^ab^	0.217	0.008
*PPARγ*	1.00 ± 0.04^b^	2.75 ± 0.39^ab^	4.87 ± 2.08^a^	2.15 ± 0.28^ab^	0.291	0.081
*FATP5*	1.00 ± 0.12^a^	0.58 ± 0.09^b^	0.55 ± 0.09^b^	0.50 ± 0.07^b^	0.002	0.002
Synthesis and storage						
*FAS*	1.00 ± 0.09^b^	1.41 ± 0.21^ab^	2.02 ± 0.40^a^	1.42 ± 0.31^ab^	0.166	0.087
*ACS*	1.00 ± 0.14	0.89 ± 0.04	0.82 ± 0.12	0.90 ± 0.03	0.360	0.408
*ACC*	1.00 ± 0.11	1.24 ± 0.11	1.33 ± 0.17	1.30 ± 0.27	0.206	0.343
*AGPAT*	1.00 ± 0.06	1.15 ± 0.05	1.07 ± 0.18	0.99 ± 0.07	0.789	0.521
*PAP*	1.00 ± 0.10^ab^	1.04 ± 0.08^ab^	0.84 ± 0.09^b^	1.29 ± 0.17^a^	0.237	0.130
*DGAT*	1.00 ± 0.18	1.17 ± 0.10	1.41 ± 0.18	1.08 ± 0.06	0.472	0.171
*GPAT*	1.00 ± 0.04^b^	1.10 ± 0.21^b^	1.53 ± 0.13^a^	0.85 ± 0.08^b^	0.977	0.051
Decomposition and release						
*CPT1*	1.00 ± 0.11	1.73 ± 0.32	1.44 ± 0.37	1.14 ± 0.17	0.927	0.163
*ATGL*	1.00 ± 0.21^b^	1.21 ± 0.15^b^	4.02 ± 0.77^a^	0.92 ± 0.09^b^	0.395	0.021
*HSL*	1.00 ± 0.15^b^	1.67 ± 0.32^b^	5.79 ± 1.08^a^	1.17 ± 0.10^b^	0.298	0.008
*MAGL*	1.00 ± 0.10^b^	1.83 ± 0.36^a^	1.22 ± 0.22^ab^	1.21 ± 0.13^ab^	0.988	0.245

All values are presented as mean ± standard error. *n* = 6; ^a,b^Different superscripts within a row are significantly different (*p* < 0.05).

The mRNA expression of sterol regulatory element binding protein 1 (*SREBP1*) in liver was decreased linearly (*p* < 0.05) and quadratically (*p* < 0.05) with the decrease of dietary n-6/n-3 PUFA ratio (Table 8). With the decrease of dietary n-6/n-3 PUFA ratio, the mRNA expression of ATP citrate lyase (*ACLY*) (linear, *p* < 0.05; quadratic, *p* < 0.05), *GPAT* (linear, *p* < 0.01; quadratic, *p* < 0.05), *ATGL* (linear, *p* < 0.05; quadratic, *p* < 0.05), *MAGL* (linear, *p* < 0.10; quadratic, *p* < 0.01) and *FATP5* (linear, *p* < 0.05; quadratic, *p* < 0.05), *PAP* (linear, *p* < 0.10), *AGPAT* (quadratic, *p* < 0.10) and *CPT1* (quadratic, *p* < 0.01) in liver was increased. Compared with the other treatment groups, pigs fed diet with n-6/n-3 PUFA ratio of 1.5:1 had the highest mRNA expression of *ACLY*, *GPAT*, *FATP2* and *FATP5* in liver (*p* < 0.05). Compared with the other three treatment groups, pigs fed diet with n-6/n-3 PUFA ratio of 5:1 had the highest mRNA expression of acyl CoA synthetase (*ACS*), *CPT1*, *GPAT*, *MAGL* and *FABP1* in liver (*p* < 0.05). In contrast, the mRNA expression of *ATGL* was significantly higher and the mRNA expression of *SREBP1* was significantly lower in pigs fed diets with n-6/n-3 PUFA ratios of 3:1 and 1.5:1 (*p* < 0.05) than that of 10:1.

**Table 8:** Effect of different n-6/n-3 PUFA ratio on gene expression related to fatty acid metabolism in liver.

Item	n-6:n-3 PUFA ratio				*p*-value	
	10:1	5:1	3:1	1.5:1	Linear	Quadratic
Absorption and transportation						
*CD36*	1.00 ± 0.10	0.71 ± 0.12	0.98 ± 0.25	0.93 ± 0.18	0.937	0.798
*FATP2*	1.00 ± 0.12^b^	0.96 ± 0.13^b^	0.74 ± 0.16^b^	1.44 ± 0.16^a^	0.157	0.027
*FATP5*	1.00 ± 0.16^b^	0.87 ± 0.10^b^	0.97 ± 0.16^b^	1.47 ± 0.17^a^	0.045	0.018
*FABP1*	1.00 ± 0.10^b^	1.84 ± 0.35^a^	1.28 ± 0.11^ab^	1.69 ± 0.28^ab^	0.210	0.334
*PPARγ*	1.00 ± 0.24	0.90 ± 0.24	0.56 ± 0.08	0.66 ± 0.10	0.101	0.230
*PPARα*	1.00 ± 0.09	1.30 ± 0.51	1.66 ± 0.35	1.39 ± 0.19	0.301	0.401
*SREBP1*	1.00 ± 0.24^a^	0.57 ± 0.14^ab^	0.48 ± 0.06^b^	0.55 ± 0.07^b^	0.041	0.031
Synthesis and storage						
*ACC*	1.00 ± 0.10	0.94 ± 0.25	1.44 ± 0.08	1.23 ± 0.35	0.244	0.488
*ACS*	1.00 ± 0.17^b^	1.80 ± 0.41^a^	0.88 ± 0.07^b^	0.98 ± 0.15^b^	0.418	0.310
*FAS*	1.00 ± 0.12^ab^	1.05 ± 0.28^ab^	1.39 ± 0.19^a^	0.68 ± 0.17^b^	0.525	0.161
*ACLY*	1.00 ± 0.10^b^	0.86 ± 0.15^b^	0.96 ± 0.17^b^	1.55 ± 0.22^a^	0.038	0.013
*PAP*	1.00 ± 0.10	1.61 ± 0.46	1.84 ± 0.45	1.98 ± 0.49	0.086	0.200
*AGPAT*	1.00 ± 0.07^b^	1.43 ± 0.12^ab^	1.98 ± 0.51^a^	1.40 ± 0.12^ab^	0.188	0.087
*GPAT*	1.00 ± 0.26^b^	3.00 ± 0.45^a^	1.83 ± 0.28^b^	3.46 ± 0.44^a^	0.005	0.021
*DGAT*	1.00 ± 0.05	1.25 ± 0.36	1.45 ± 0.41	1.48 ± 0.10	0.184	0.388
Decomposition and release						
*CPT1*	1.00 ± 0.08^b^	3.41 ± 0.65^a^	2.79 ± 0.39^a^	2.45 ± 0.36^a^	0.124	0.006
*ATGL*	1.00 ± 0.13^b^	1.19 ± 0.16^ab^	1.47 ± 0.14^a^	1.43 ± 0.10^a^	0.016	0.042
*HSL*	1.00 ± 0.13	1.12 ± 0.10	1.43 ± 0.18	1.11 ± 0.19	0.386	0.272
*MAGL*	1.00 ± 0.08^b^	2.10 ± 0.26^a^	1.79 ± 0.11^a^	1.75 ± 0.19^a^	0.061	0.004

All values are presented as mean ± standard error. *n* = 6; ^a,b^Different superscripts within a row are significantly different (*p* < 0.05).

The mRNA expression of *CD36* (linear, *p* < 0.001; quadratic, *p* < 0.01), *FABP4* (linear, *p* < 0.01; quadratic, *p* < 0.001), *PPARγ* (quadratic, *p* < 0.05), acetyl CoA carboxylase (*ACC*) (linear, *p* < 0.01; quadratic, *p* < 0.01), *ACS* (linear, *p* < 0.10), *ACLY* (linear, *p* < 0.001; quadratic, *p* < 0.001), *AGPAT* (linear, *p* < 0.05; quadratic, *p* < 0.01), *GPAT* (linear, *p* < 0.10), *DGAT* (quadratic, *p* < 0.01), *CPT1* (linear, *p* < 0.05; quadratic, *p* < 0.10) and *HSL* (linear, *p* < 0.01; quadratic, *p* < 0.001) in gastrocnemius muscle was decreased with decreasing ratios of dietary n-6/n-3 PUFA (Table 9). With the decreased ratios of dietary n-6/n-3 PUFA, the mRNA expression of *FABP1* (linear, *p* < 0.05; quadratic, *p* < 0.05), *FATP4* (linear, *p* < 0.05; quadratic, *p* < 0.10), *PAP* (quadratic, *p* < 0.10) and *MAGL* (quadratic, *p* < 0.01) in gastrocnemius muscle was increased. Pigs fed diets with n-6/n-3 PUFA ratio of 5:1 had the lowest mRNA expression of *FABP4*, *PPARγ*, *AGPAT*, *GPAT*, *ATGL*, and *HSL* (*p* < 0.05) in gastrocnemius muscle compared with diets with n-6/n-3 PUFA ratios of 10:1, 3:1 and 1.5:1. Pigs fed diet with n-6/n-3 PUFA ratio of 3:1 had the highest mRNA expression of *FATP1* and *MAGL* (*p* < 0.05) and the lowest mRNA expression of *ACC* and *DGAT* (*p* < 0.05) in gastrocnemius muscle compared with other treatment groups. Compared with diets with n-6/n-3 PUFA ratios of 10:1, 5:1 and 3:1, pigs fed diets with n-6/n-3 PUFA ratio of 1.5:1 had the lowest mRNA expression of *CD36* and *ACLY* in gastrocnemius muscle (*p* < 0.05).

**Table 9:** Effect of different n-6/n-3 PUFA ratio on gene expression related to fatty acid metabolism in gastrocnemius muscle.

Item	n-6:n-3 PUFA ratio				*p*-value	
	10:1	5:1	3:1	1.5:1	Linear	Quadratic
Absorption and transportation						
*CD36*	1.00 ± 0.07^a^	0.83 ± 0.11^ab^	0.76 ± 0.05^bc^	0.59 ± 0.04^c^	<0.001	0.002
*FABP1*	1.00 ± 0.18^bc^	0.91 ± 0.09^c^	1.86 ± 0.16^a^	1.34 ± 0.09^b^	0.025	0.044
*FATP4*	1.00 ± 0.16	0.92 ± 0.14	1.16 ± 0.26	2.53 ± 0.98	0.050	0.057
*FABP4*	1.00 ± 0.06^a^	0.61 ± 0.07^b^	0.64 ± 0.05^b^	0.65 ± 0.05^b^	0.005	<0.001
*PPARγ*	1.00 ± 0.12^a^	0.60 ± 0.09^b^	0.63 ± 0.08^b^	0.73 ± 0.10^ab^	0.123	0.020
*SREBP1*	1.00 ± 0.15	1.01 ± 0.22	1.13 ± 0.28	1.20 ± 0.30	0.488	0.784
Synthesis and storage						
*ACC*	1.00 ± 0.07^a^	0.66 ± 0.16^b^	0.55 ± 0.05^b^	0.60 ± 0.08^b^	0.010	0.006
*ACS*	1.00 ± 0.13	0.75 ± 0.10	0.86 ± 0.06	0.70 ± 0.10	0.094	0.231
*FAS*	1.00 ± 0.08^ab^	0.75 ± 0.06^b^	1.44 ± 0.36^a^	0.84 ± 0.08^b^	0.816	0.710
*ACLY*	1.00 ± 0.05^a^	0.63 ± 0.08^b^	0.62 ± 0.09^b^	0.51 ± 0.05^b^	<0.001	<0.001
*PAP*	1.00 ± 0.18^ab^	1.28 ± 0.26^ab^	1.72 ± 0.46^a^	0.61 ± 0.05^b^	0.607	0.065
*AGPAT*	1.00 ± 0.07^a^	0.56 ± 0.09^b^	0.63 ± 0.05^b^	0.66 ± 0.06^b^	0.019	0.001
*GPAT*	1.00 ± 0.15^a^	0.57 ± 0.10^b^	0.77 ± 0.11^ab^	0.58 ± 0.08^b^	0.066	0.119
*DGAT*	1.00 ± 0.09^ab^	0.65 ± 0.09^b^	0.57 ± 0.05^b^	1.39 ± 0.35^a^	0.275	0.009
Decomposition and release						
*CPT1*	1.00 ± 0.08	0.88 ± 0.12	0.74 ± 0.08	0.69 ± 0.11	0.021	0.070
*ATGL*	1.00 ± 0.07^a^	0.60 ± 0.07^b^	0.93 ± 0.15^a^	0.78 ± 0.10^ab^	0.504	0.453
*HSL*	1.00 ± 0.06^a^	0.35 ± 0.06^b^	0.47 ± 0.06^b^	0.38 ± 0.07^b^	0.001	<0.001
*MAGL*	1.00 ± 0.10^c^	1.44 ± 0.14^ab^	1.85 ± 0.22^a^	1.04 ± 0.07^bc^	0.562	0.001

All values are presented as mean ± standard error. *n* = 6; ^a,b,c^Different superscripts within a row are significantly different (*p* < 0.05).

The mRNA expression of *FABP4* (linear, *p* < 0.01; quadratic, *p* < 0.01), *PPARγ* (linear, *p* < 0.05; quadratic, *p* < 0.05), *ACC* (linear, *p* < 0.05; quadratic, *p* < 0.05), *CPT1* (linear, *p* < 0.05; quadratic, *p* < 0.10), *PAP* (linear, *p* < 0.05; quadratic, *p* < 0.05), *AGPAT* (linear, *p* < 0.05; quadratic, *p* < 0.05), *GPAT* (linear, *p* < 0.01; quadratic, *p* < 0.05), *ATGL* (linear, *p* < 0.05) and *MAGL* (linear, *p* < 0.05; quadratic, *p* < 0.10) was increased with decreasing ratios of dietary n-6/n-3 PUFA (Table 10). With the decreased ratios of dietary n-6/n-3 PUFA, the mRNA expression of *ACS* (*p* < 0.05), *ACLY* (*p* < 0.10), *HSL* (*p* < 0.05), *CD36* (*p* < 0.05), and lipoprotein lipase (*LPL*) (*p* < 0.05) in gastrocnemius muscle was increased quadratically. Compared with diets with n-6/n-3 PUFA ratios of 10:1, 3:1 and 1.5:1, pigs fed diets with n-6/n-3 PUFA ratios of 5:1 had the highest mRNA expression of *SREBP1* (*p* < 0.10), *ACLY* (*p* < 0.05) and *AGPAT* (*p* < 0.01) in gastrocnemius muscle. Pigs fed diets with n-6/n-3 PUFA ratios of 3:1 had the highest mRNA expression of *CD36*, *LPL*, *ACS*, *PAP* and *HSL* (*p* < 0.05) in subcutaneous fat compared with diets with n-6/n-3 PUFA ratios of 10:1, 5:1 and 1.5:1. The group of diets with n-6/n-3 PUFA ratios of 1.5:1 had the highest mRNA expression of *FABP4*, *PPARγ*, *ACC*, *GPAT*, *ATGL* and *MAGL* (*p* < 0.05) in subcutaneous fat compared with other treatments.

**Table 10:** Effect of different n-6/n-3 PUFA ratio on gene expression related to fatty acid metabolism in subcutaneous fat.

Item	n-6:n-3 PUFA ratio				*p*-value	
	10:1	5:1	3:1	1.5:1	Linear	Quadratic
Absorption and transportation						
*CD36*	1.00 ± 0.08^ab^	1.03 ± 0.04^ab^	1.14 ± 0.09^a^	0.81 ± 0.08^b^	0.244	0.046
*FATP4*	1.00 ± 0.21	0.76 ± 0.04	0.61 ± 0.02	0.81 ± 0.11	0.226	0.101
*FABP4*	1.00 ± 0.04^b^	2.08 ± 0.34^a^	1.85 ± 0.16^a^	2.27 ± 0.16^a^	0.002	0.003
*LPL*	1.00 ± 0.14^b^	1.42 ± 0.10^ab^	1.65 ± 0.11^a^	1.17 ± 0.20^b^	0.339	0.010
*PPARγ*	1.00 ± 0.05^b^	1.28 ± 0.11^b^	1.05 ± 0.11^b^	1.67 ± 0.19^a^	0.010	0.019
*SREBP1*	1.00 ± 0.09^b^	1.50 ± 0.13^a^	1.24 ± 0.12^ab^	1.23 ± 0.13^ab^	0.493	0.127
Synthesis and storage						
*ACC*	1.00 ± 0.06^b^	2.68 ± 0.42^a^	2.43 ± 0.43^a^	2.76 ± 0.45^a^	0.011	0.010
*ACS*	1.00 ± 0.13^b^	1.21 ± 0.08^ab^	1.54 ± 0.13^a^	1.00 ± 0.12^b^	0.622	0.020
*FAS*	1.00 ± 0.24	0.87 ± 0.26	0.98 ± 0.29	0.76 ± 0.12	0.555	0.829
*ACLY*	1.00 ± 0.03^b^	2.18 ± 0.48^a^	1.30 ± 0.16^b^	1.08 ± 0.12^b^	0.657	0.068
*PAP*	1.00 ± 0.04^b^	1.42 ± 0.09^a^	1.42 ± 0.14^a^	1.38 ± 0.11^a^	0.032	0.011
*AGPAT*	1.00 ± 0.07^b^	1.61 ± 0.10^a^	1.46 ± 0.19^a^	1.52 ± 0.08^a^	0.030	0.012
*GPAT*	1.00 ± 0.02^b^	1.62 ± 0.23^a^	1.23 ± 0.07^b^	1.82 ± 0.07^a^	0.006	0.026
*DGAT*	1.00 ± 0.10^b^	1.33 ± 0.08^a^	1.21 ± 0.12^ab^	1.28 ± 0.06^ab^	0.115	0.127
Decomposition and release						
*CPT1*	1.00 ± 0.05^b^	1.16 ± 0.13^ab^	1.21 ± 0.14^ab^	1.42 ± 0.15^a^	0.022	0.076
*ATGL*	1.00 ± 0.09^b^	1.35 ± 0.09^a^	1.18 ± 0.14^ab^	1.40 ± 0.07^a^	0.113	0.045
*HSL*	1.00 ± 0.04^b^	1.45 ± 0.05^a^	1.60 ± 0.27^a^	1.32 ± 0.07^ab^	0.123	0.017
*MAGL*	1.00 ± 0.04^b^	1.43 ± 0.18^ab^	1.29 ± 0.09^ab^	1.60 ± 0.21^a^	0.022	0.072

All values are presented as mean ± standard error. *n* = 6; ^a,b^Different superscripts within a row are significantly different (*p* < 0.05).

### Effect of different n-6/n-3 PUFA ratios on the differentially expressed genes associated with fatty acid metabolism in different tissues

**Figure 1:**
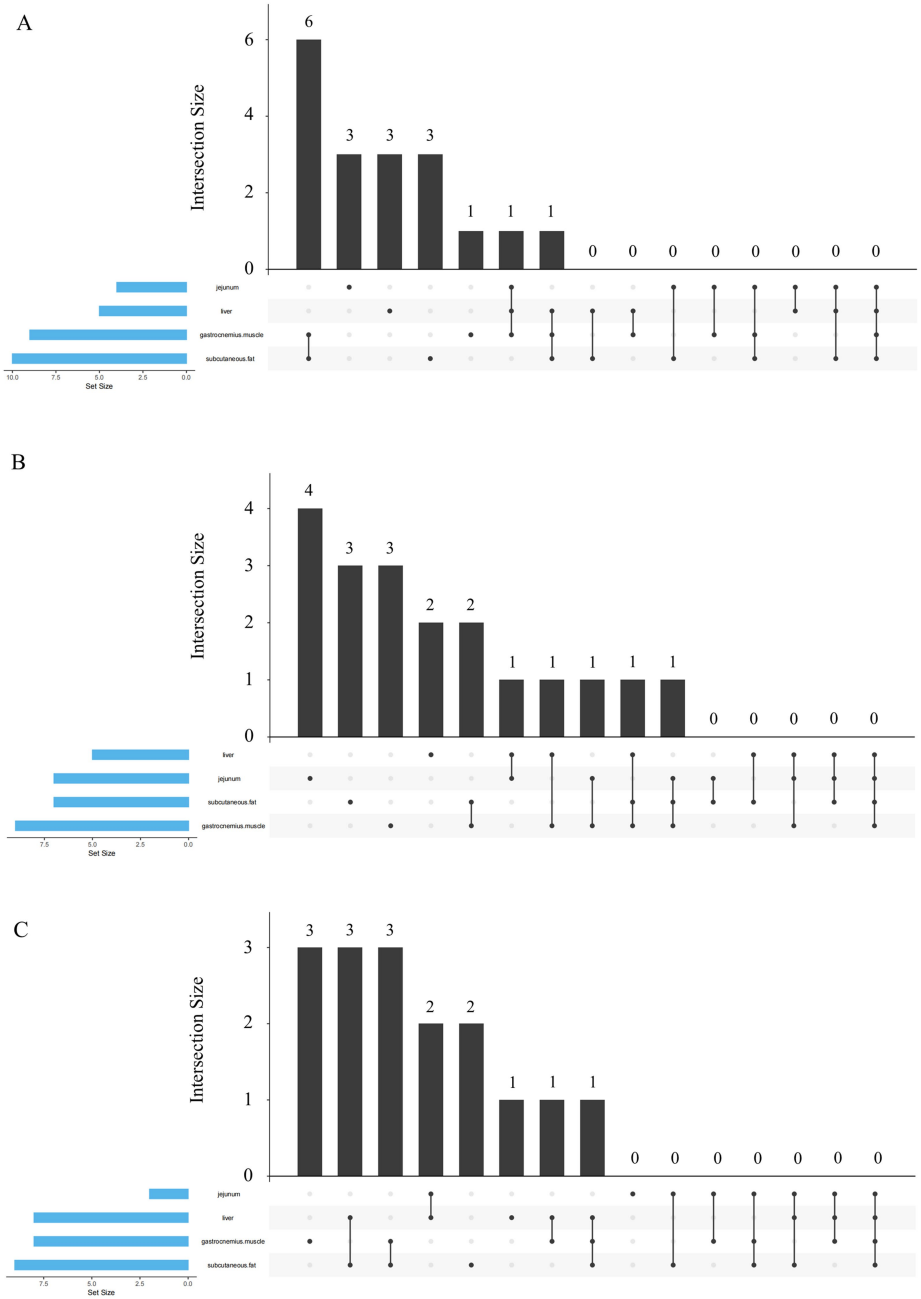
Upset plot analysis of the differentially expressed genes associated with fatty acid metabolism in different tissues. **(A)** Upset plot analysis of treatment groups with dietary n-6/n-3 PUFA ratios of 10 and 5. **(B)** Upset plot analysis of treatment groups with dietary n-6/n-3 PUFA ratios of 10 and 3. **(C)** Upset plot analysis of treatment groups with dietary n-6/n-3 PUFA ratios of 10 and 1.5.

Figure 1A illustrates the differentially expressed genes (DEGs) associated with fatty acid metabolism in response to dietary n-6/n-3 PUFA ratios of 10 and 5 across four tissues: subcutaneous fat (10 DEGs), gastrocnemius muscle (9 DEGs), jejunum (5 DEGs), and liver (4 DEGs). Among these, six DEGs were shared between subcutaneous fat and gastrocnemius muscle. Tissue-specific DEGs included three in the jejunum, three in the liver, and three in subcutaneous fat, while one was unique to the gastrocnemius muscle. Additionally, one DEG was common to gastrocnemius muscle, liver, and jejunum; one to subcutaneous fat, gastrocnemius muscle, and liver; one to subcutaneous fat, gastrocnemius muscle, and jejunum; and one to subcutaneous fat, gastrocnemius muscle, and liver. In Figure 1B, treatment groups with n-6/n-3 PUFA ratios of 10 and 3 exhibited DEGs in gastrocnemius muscle (7), subcutaneous fat (9), jejunum (7), and liver (5). 4 DEGs were specific to the jejunum, three to subcutaneous fat, 3 to gastrocnemius muscle, and 2 to the liver. Shared DEGs included two between subcutaneous fat and gastrocnemius muscle, one between liver and jejunum, one among liver, jejunum, and gastrocnemius muscle (repeated twice), and one among subcutaneous fat, gastrocnemius muscle, and liver. Figure 1C displays DEGs in response to n-6/n-3 PUFA ratios of 10 and 1.5, with subcutaneous fat (9 DEGs), gastrocnemius muscle (8 DEGs), liver (8 DEGs), and jejunum (2 DEGs) showing differential expression. Three DEGs were unique to gastrocnemius muscle, three were shared between subcutaneous fat and liver, and three between subcutaneous fat and gastrocnemius muscle. Two DEGs were common to liver and jejunum, two were liver-specific, and one was gastrocnemius muscle-specific. Additionally, one DEG was shared between liver and gastrocnemius muscle, and one among subcutaneous fat, gastrocnemius muscle, and liver.

## Discussion

The role of dietary n-6 and n-3 PUFA balance in the growth and development (1), heredity (23, 24) and resistance to inflammation (25) of livestock and poultry has been paid more and more attention by scientists. Furthermore, n-6 and n-3 PUFA have important regulatory effects on the metabolism of fatty acids in livestock and poultry. The metabolism of fatty acids in different tissues and organs of animals at different growth stages is also obvious different (26). Therefore, we mainly aimed to investigate the effects of dietary n-6/n-3 PUFA ratio on growth performance and fatty acid metabolism in intestine, liver, muscle and adipose tissue of nursery pigs. Our previous studies demonstrated that the fatty acid profiles of intestinal mucosa, liver, and muscle tissue of nursery pigs largely mirrored the composition of dietary fatty acids (27–29). After 4 weeks of feeding, growth performance of pigs fed diets with n-6/n-3 PUFA ratios of 1.5:1 and 3:1 was significantly improved, which is indicated by higher ADG and lower F/G ratio. Our results are in line with the observations by Doaa et al. that chickens fed a diet with 1:1 ratio of sunflower oil to linseed oil (dietary n-6/n-3 PUFA ratio of 3:1) had the optimum growth performance (25). Previous studies have shown that lower ratios of n-6/n-3 PUFA (i.e., 1.5:1–5:1) promoted the absorption and utilization of fatty acids and free amino acids, and improved muscle and fat composition of finishing pigs (18). Our data suggest that lower dietary ratios of n-6 and n-3 PUFA (i.e., 1.5:1–3:1) are more beneficial to pig growth.

The contents of blood lipids such as TC, TG, HDL and LDL are closely related to the absorption and transport of fatty acids in the diet, and are also important indexes indicating the health status of the body (30–32). Our data showed that the contents of TC, TG, HDL and LDL in serum of pigs fed diet with n-6/n-3 PUFA ratio of 1.5:1 were markedly reduced compared with the other treatment groups. Consistent with our findings, Liu et al. and Doaa et al. reported that lowering n-6/n-3 PUFA ratios in diets could significantly reduce serum TC, TG and LDL contents in pigs (33), chickens (25) and ducks (34). Wang et al. showed that the contents of blood lipids and the composition of long-chain fatty acids in serum changed with the variation of dietary n-6/n-3 PUFA ratios (20). This indicates that blood lipids are closely related to dietary lipid composition. In addition, insulin, adiponectin and leptin in the blood are not only key hormones that regulate blood lipids, but also are affected by the intake of n-3 PUFA (35, 36). Our results demonstrated that pigs fed diet with n-6/n-3 PUFA ratio of 3:1 had the highest concentrations of insulin, adiponectin and leptin in the serum compared with diets with the other treatment groups. However, the blood glucose level of pigs was not affected by dietary n-6/n-3 PUFA ratios. It is well known that blood glucose level is regulated not only by insulin but also by counter-regulatory hormones such as glucagon, adrenaline, cortisol, and growth hormone, which collectively maintain glucose homeostasis (37). Therefore, we propose that the combined influence of these counter-regulatory hormones might counteracts insulin’s glucose-reducing effects. Fan et al. found that the contents of blood adiponectin and leptin of pigs fed diet supplemented with Mulberry leaf increased, while the concentrations of serum TG and TC decreased (38). Lower n-6/n-3 PUFA ratios mean higher levels of n-3 PUFA in the diet. A large number of studies have shown that n-3 PUFA have the effect of lowering blood lipids (32, 39). The above results show that reducing dietary n-6/n-3 PUFA ratios (i.e., 1.5:1) is more advantageous to reduce blood lipid levels.

The contents of n-6 and n-3 PUFA in the diet can directly affect the metabolism of fatty acids in the body, including the absorption and transportation, synthesis and storage, decomposition and release of fatty acids (11). The small intestine is the main place for the digestion and absorption of fatty acids, so we first researched the metabolism of fatty acids in jejunum. The results showed that mRNA expression of fatty acid intake and transport-related genes such as *FATP2* and *FATP5* in jejunum was significantly decreased with the decrease of dietary n-6/n-3 PUFA ratio, while mRNA expression of fatty acid decomposition and release-related genes (*ATGL*, *HSL* and *MAGL*) was significantly increased. ATGL and HSL are the main novel triglyceride lipases in animals and are important targets for regulating fat deposition and improving meat quality (40, 41). FATP, a family of membrane binding proteins, can catalyze the ATP-dependent esterification of long-chain fatty acids to their acyl derivatives, and has been shown to stimulate fatty acid intake and transport (42). We also observed the same result in gastrocnemius muscle. With the decrease of n-6/n-3 PUFA ratio in the diet, mRNA expression levels of genes associated with fatty acid intake and transport (*CD36*, *FABP4*, and *PPARγ*) and synthesis and storage (*ACC*, *ACLY*, *PAP*, *GPAT*, and *DGAT*) in gastrocnemius muscle were significantly decreased, while mRNA expression of decomposition and release related genes (*MAGL*) was increased. Li et al. found that the expression levels of *FATP1* and *FATP4* were decreased in skeletal muscle of pigs fed diets with n-6/n-3PUFA ratios of 3:1 and 1:1 (18). A balanced n-6/n-3 PUFA ratio in the diet is linked to improving body composition, and reducing intermuscular, visceral and subcutaneous fat while preserving lean muscle mass, thereby enhancing overall muscle quality (43–45). In agreement with our results, Nong et al. showed that the reduction of the dietary n-6/n-3 PUFA ratio could promote the expression of *HSL* and *CPT1* in longissimus dorsi muscle of Heigai pigs (19). According to our data, we demonstrate that reducing the dietary n-6/n-3 PUFA ratios (i.e., 1.5:1–5:1) inhibits the intake of fatty acids to intestine and muscle tissue, and promotes the breakdown of these fatty acids.

The liver is the main site of oxidative breakdown of fatty acids. Further analysis showed that mRNA expression levels of genes associated with intake and transport (*FATP2*, *FATP5*, and *FABP1*), synthesis and storage (*ACLY* and *GPAT*), and decomposition and release (*CPT1*, *ATGL*, and *MAGL*) of fatty acids in liver were obviously increased with the reduction of n-6/n-3 PUFA ratio in the diet. Dierge et al. demonstrated that treatment with either n-6 or n-3 PUFAs alone increased free fatty acid accumulation in lipid droplets across different cell types (46). Our study revealed that subcutaneous fat exhibited the highest number of DEGs associated with fatty acid metabolism across all treatment groups. This finding aligns with the well-established role that adipose tissue is the central organ of fat synthesis and deposition (47). The results showed that mRNA expression levels of genes related to intake and transport (*CD36*, *FABP4*, *LPL*, *PPARγ*, and *SREBP1*), synthesis and storage (*ACC*, *ACS*, *ACLY*, *PAP*, *AGPAT*, *GPAT*, and *DGAT*), and decomposition and release (*CPT1*, *ATGL*, *HSL*, and *MAGL*) of fatty acids in subcutaneous fat were significantly increased with the reduction of n-6/n-3 PUFA ratio in the diet. These observations can be supported by Shan and colleagues who found that the mRNA expression levels of pATGL and HSL were higher in the subcutaneous adipose tissue of lean-type pigs compared with fat-type pigs (41). Diets with lower n-6/n-3 PUFA ratios increased the expression of genes related to decomposition and release of fatty acids in subcutaneous fat, thereby promoting the decomposition and release of lipids in adipose tissue. CD36 binds to long-chain PUFA to regulate PPARγ transcription and then up-regulate FABP4 expression, thus playing an important role in lipid uptake and metabolic homeostasis regulation in adipose tissue (48). In addition, the increase of FATP1 accelerates the rate of long-chain fatty acid transport and channel, but does not increase liver lipid accumulation (49). More importantly, FATP4 plays an important role in regulating lipid metabolism as a fatty cyl-CoA synthetase (50). Consistent with our results, Nong et al. showed that reducing dietary n-6/n-3 PUFA ratio increased the levels of *FABP4*, *HSL* and *CPT1* in subcutaneous adipose tissue of Heigai pigs (19). Therefore, it is suggested that diets with lower n-6/n-3 PUFA ratios (i.e., 1.5:1–5:1) can promote the metabolism of fat in liver and adipose tissue of pigs.

In our research, the n-3/n-6 PUFA balance not only improved the growth performance of nursery pigs but also modulated lipid metabolism. Lipid metabolism plays a crucial role in regulating the growth performance of pigs through its dual effects on energy partitioning and endocrine regulation. From an energetic perspective, enhanced intestinal lipid absorption and transport facilitate the release of lipids into circulation, which are subsequently delivered to hepatic and adipose tissues. This metabolic shift promotes lipolysis and fat mobilization, thereby redirecting energy substrates toward muscle tissue rather than adipose deposition. Concurrently, lipid metabolism influences endocrine signaling pathways that modulate growth. Insulin stimulates muscle protein synthesis, while adipokines such as leptin and adiponectin function to suppress excessive fat accumulation. These coordinated mechanisms collectively contribute to improved growth performance in pig.

## Conclusion

In conclusion, diets with lower n-6/n-3 PUFA ratios improve growth performance, reduce blood lipids, facilitate lipid metabolism in intestine, liver and subcutaneous fat, and inhibit fatty acid absorption, synthesis and storage in gastrocnemius muscle in nursery pigs. The dietary n-6/n-3 PUFA ratio of 1.5:1 appears optimal for growth performance. Nevertheless, when considering cost effectiveness, the ratio of 3:1 may represent a more practical choice. This finding has a good reference value for formulating nursery pig feed with appropriate ratio of n-6/n-3 PUFA.

## Data Availability

The original contributions presented in the study are included in the article/supplementary material, further inquiries can be directed to the corresponding author.

## Glossary

ACC: Acetyl CoA carboxylase
ACLY: ATP citrate lyase
ACS: Acyl CoA synthetase
ADFI: Average daily feed intake
ADG: Average daily gain
AGPAT: Acylglycerol 3 phosphate acyltransferase
ATGL: Adipose triglyceride lipase
CPT1: Carnitine palmitoyl transferase 1
DGAT: Diacylglycerol acyltransferases
F/G: Feed to gain ratio
FABP: Fatty acid-binding protein
FAT/CD36: Fatty acid translocase
FATP: Fatty acid transport protein
G3P: Glycerol 3 phosphate
GPAT: Glycerol 3 phosphate acyltransferase
HDL: High density lipoprotein
HSL: Hormone sensitive lipase
LDL: Low density lipoprotein
MAGL: Monoacylglycerol lipase
MGAT: Monoacylglycerol acyltransferase
PAP: Phosphatidic acid phosphatase
PPAR: Peroxisome proliferator activated receptor
PUFA: Polyunsaturated fatty acid
SREBP1: Sterol regulatory element binding protein 1
TAG: Triglycerides
TC: Total cholesterol
TG: Triglyceride
