# Correction: Effects of dietary n-6/n-3 PUFA ratio on growth performance and lipid metabolism in nursery pigs

**DOI:** 10.3389/fvets.2025.1729211

**Published:** 2025-11-06

**Authors:** Junjie Guo, Xiaoqian Chen, Huiling Zhu, Kan Xiao, Yanbing Zhang, Shiwei Zhao, Guoshun Chen, Yulan Liu

**Affiliations:** 1 College of Animal Science and Technology, Gansu Agricultural University, Lanzhou, China; 2 Hubei Key Laboratory of Animal Nutrition and Feed Science, Wuhan Polytechnic University, Wuhan, China; 3 Shandong Zhongmu Feed Technology Co., Ltd., Binzhou, Shandong, China; 4 Shandong Crelipids Biotechnology Co., Ltd., Binzhou, Shandong, China

**Keywords:** n-6 PUFA, n-3 PUFA, fatty acid, lipid metabolism, nursery pigs

Affiliation 1, College of Animal Science and Technology, Gansu Agricultural University, Lanzhou, China was omitted from the paper. This affiliation has now been added for author Yulan Liu.

The original version of this article has been updated.

